# Outcomes After Open Surgical, Hybrid, and Endovascular Revascularization for Acute Limb Ischemia

**DOI:** 10.1177/15266028231210232

**Published:** 2023-11-27

**Authors:** Nikolaos Konstantinou, Angeliki Argyriou, Felicitas Dammer, Theodosios Bisdas, Gregory Chlouverakis, Giovanni Torsello, Nikolaos Tsilimparis, Konstantinos Stavroulakis

**Affiliations:** 1 Department of Vascular Surgery, University Hospital LMU Munich, Munich, Germany; 2 Department of Vascular Surgery, Marien Hospital Herne, Herne, Germany; 3 Department of Vascular and Endovascular Surgery, Athens Medical Center, Athens, Greece; 4 Biostatistics Laboratory, Department of Social Medicine, School of Medicine, University of Crete, Crete, Greece; 5 Department of Vascular Surgery, St. Franziskus Hospital GmbH, Muenster, Germany

**Keywords:** acute limb ischemia, open surgery, hybrid surgery, revascularization, stent occlusion, bypass occlusion

## Abstract

**Purpose:**

To report the performance of surgical treatment (ST), hybrid treatment (HT), and endovascular treatment (ET) for patients with acute limb ischemia (ALI).

**Methods:**

This is a retrospective, comparative study of all consecutive patients with ALI treated in 2 tertiary centers between April 2010 and April 2020. Amputation and/or death (amputation-free survival; AFS) was the primary composite endpoint. Mortality, major amputation, and reintervention during follow-up were additionally analyzed. Proportional hazards modeling was used to identify confounders, results are presented as hazard ratio (HR) and 95% confidence intervals (CIs).

**Results:**

In total, 395 patients (mean age=71.1±13.6 years; 51.1% female) were treated during the study period. Surgical treatment was preferred in 150 patients (38%), while 98 were treated by HT (24.8%) and 147 by ET (37.2%). Rutherford class IIa was the most common clinical presentation in the ET group (50.3%), whereas Rutherford IIb was most common in the ST (54%) and HT (48%) groups (p<0.001). Significantly, more patients presented with a de novo lesion in the ST and HT groups (79.3% and 64.3%, respectively) compared with ET (53.7%; p<0.001). Median follow-up was 20 months (range=0–111 months). In the multivariate analysis, ET showed significantly better AFS during follow-up compared with ST (HR=1.89, 95% CI=1.2–2.9, p<0.001) and HT (HR=1.73, 95% CI=1.1–3.1, p<0.001). Mortality during follow-up was also significantly lower after ET compared with ST (HR=2.21, 95% CI=1.31–3.74, p=0.003) and HT (HR=2.04, 95% CI=1.17–3.56, p=0.012). Endovascular treatment was associated with lower amputation rate compared with ST (HR=2.27, 95% CI=1.19–4.35, p=0.013) but was comparable with HT (HR=2.00, 95% CI=0.98–4.06, p=0.055). Reintervention rates did not differ significantly between the groups (ET vs ST: HR=1.52, 95% CI=0.99–2.31, p=0.053; ET vs HT: HR=1.3, 95% CI=0.81–2.07, p=0.27).

**Conclusion:**

Endovascular treatment for ALI was associated with improved AFS and comparable reintervention rates compared with open surgical and hybrid therapy.

**Clinical Impact:**

Treatment of acute lower limb ischemia remains a challenge for clinicians with high morbidity and mortality rates. Endovascular revascularization is considered first line treatment for many and hybrid treatments are becoming more common, however data is limited to either old trials, small series or with short follow-up. We present herein our 10-year experience with all available devices and techniques for open surgical, endovascular and hybrid acute limb ischemia treatments and compare their outcomes.

## Introduction

Acute limb ischemia (ALI) is one of the most common emergencies in vascular medicine with an estimated incidence of 22 to 26 per 100 000 patients per year.^1^ In addition, it is potentially one of the most debilitating conditions, with amputation rates of up to 40% and mortality ranging from 15% to 20%.^2^ Therefore, the need for prompt treatment and the development of treatment algorithms remains crucial.

Historically, surgical treatment (ST) was considered the treatment of choice given the need for immediate revascularization and reperfusion of the affected limb. A shift toward endovascular procedures began when large, randomized trials from the 1990s showed that catheter-directed thrombolysis (CDT) achieved similar results as ST in ALI patients, while in many cases avoiding surgery altogether.^3–5^ However, the positive results were mitigated by the risk of major, life-threatening bleeding in thrombolysis patients, such as intracranial bleeding.^6,7^ Thus, many physicians still opting for a surgery-first algorithm when treating ALI patients. In addition, the treatment of CDT resisting clots might require a time-consuming therapy and is associated with delayed reperfusion as well as prolonged intensive care unit (ICU) stay.^8^

In the decades following these trials, a plethora of novel endovascular modalities, such as pharmacomechanical thrombectomy (PMT) and aspiration thrombectomy systems, as well as newer thrombolytic agents, such as alteplase (rtPA: recombinant tissue plasminogen activator) have been introduced, showing promising results.^9–11^ Besides the theoretical reduced risk of major bleeding complications, modern endovascular treatment (ET) options also enable a fast clot removal and an immediate reperfusion of the lower limb. Moreover, the combination of open surgical and endovascular procedures led to the rise of hybrid treatment (HT) modalities, leading to a recommendation in the 2020 European Society Vascular Surgery (ESVS) ALI Guidelines that ALI procedures should be performed in a theater equipped with angiographic capabilities.^12^

Despite the widespread use of hybrid and endovascular techniques in the treatment of ALI, bibliographic evidence remains sparse, limited to either small series, or cohorts with short follow-up periods.^9,13^ In addition, in most series, CDT was the primary endovascular option, while the use of thrombectomy devices was limited. The aim of this study is to compare the outcomes of open surgical, hybrid, and endovascular strategies using all currently available treatment options for patients with lower extremity ALI.

## Materials and Methods

### Study Design, Patient Selection, and Data Collection

This was a retrospective, comparative study of consecutive patients treated for ALI in 2 tertiary vascular centers between 2010 and 2020. Patient data were collected in a collaborative registry between the 2 centers using electronic patient database records and by contacting individual patients to obtain follow-up relevant information. Ethical approval was granted from the institutional review boards of both centers and the need for informed patient consent was waived due to the retrospective nature of the study.

All included patients underwent either ST, HT, or ET in a hybrid theater or angio-suite and were categorized accordingly in one of the 3 groups. Patients presenting with ALI due to occlusion of native peripheral vessels, occlusion of bypass grafts, stents, and stent-grafts were included in the study. Patients with occluded popliteal aneurysms, distal embolization during an open or endovascular procedure, vascular trauma, and aneurysm-related aortic occlusions were excluded. Bilateral acute lower limb ischemia cases were included in the database.

### Technical and Procedural Details

Both participating centers were high-volume vascular clinics with hybrid operating theaters and all treatment modalities readily available. The choice of treatment remained at the discretion of the operating physician and regarding the retrospective nature of the study, we did not perform any separate analysis regarding anatomical regions and choice of treatment. Open ST included embolectomy or thrombectomy of the occluded vessel and/or bypass grafting.

Endovascular revascularization consisted of CDT, PMT (AngioJet, Boston Scientific; Marlborough, MA), aspiration thrombectomy (Indigo Catheter, Penumbra Inc., CA), rotational thrombectomy (Rotarex, BD medical equipment; Franklin Lakes, NJ) and adjunct procedures, including angioplasty, stenting, and/or atherectomy. For CDT, a Cragg-McNamara catheter (Medtronic; Dublin, Ireland) was placed in the occluded vascular segment and continuous infusion of rtPA with a rate of 1 mg/h was initiated after an initial bolus of 6 to 8 mg. In addition, heparin was administered through the lysis catheter at 400 IU/h as well as through the sheath at a rate of 400 IU/h to avoid sheath thrombosis. No systemic anticoagulation was used. For the entire CDT duration, patients were monitored on an ICU or intermediate care unit. A control angiography was performed after 12 to 24 hours to control the efficacy of the CDT, followed by any additional procedures if needed. Plasma fibrinogen levels were not routinely controlled, as the effectiveness and predictive value of this test have not been proven.^14^ A completion angiography was always performed, including imaging of the ankle and foot vessels. Selection of the different thrombectomy devices was based on the location of the occlusion (iliac, femoropopliteal, and tibial), the nature of the disease (embolic, thrombotic, bypass, stent thrombosis, etc) and the technical skills of the treating physician.

Hybrid revascularization was defined as any combination of open surgical and endovascular techniques during a single procedure, even if the endovascular part was unplanned or used as “bail-out.” Endovascular treatment of open surgical complications during a secondary procedure was not included in the hybrid group and was considered as reintervention. Moreover, performing an angiography after bypass grafting or conventional embolectomy did not qualify them as hybrid.

### Definitions, Endpoints, and Measures of Outcomes

Digital patient records were scrutinized for demographic characteristics (age and sex), comorbidities (coronary artery disease, hypertension, chronic kidney disease smoking, malignancy, etc), and clinical and anatomical disease characteristics (duration and severity of symptoms, previous vascular interventions, etc). Malignancy was defined as active neoplastic disease in the last 6 months before the index procedure; chronic kidney disease was defined according to the revised KDIGO (Kidney Disease Improving Global Outcomes) criteria.^15^ All included baseline variables can be found in Tables 1 and 2. Patients as well as their primary care physicians were contacted in cases of missing information on baseline characteristics or missing follow-up data.

**Table 1.** Demographic Characteristics of the Study Group.

Comorbidities and cardiovascular risk factors	Surgery (n=150)	Hybrid (n=98)	Endovascular (n=147)	p	Total (n=395)
Male sex	65 (43.3%)	51 (52%)	77 (52.4%)	ns	193 (48.9%)
Age	73.1±14.8	71.3±12.8	69±12.5	0.035	
Coronary artery disease	57 (38.3%)	41 (41.4%)	49 (33.3%)	ns	147 (37.2%)
Atrial fibrillation	59 (39.6%)	37 (37.4%)	31 (21.1%)	0.001	127 (32.2%)
Malignancy	39 (26.2%)	24 (24.2%)	17 (11.6%)	0.004	80 (20.3%)
Hypertension	116 (77.3%)	79 (80.6%)	118 (80.3%)	ns	313 (79.2%)
Dyslipidemia	73 (48.7%)	42 (42.9%)	90 (61.2%)	0.011	205 (51.9%)
Smoking	54 (38.6%)	41 (43.2%)	54 (36.7%)	ns	149 (39%)
Diabetes mellitus	38 (25.3%)	26 (26.5%)	37 (25.2%)	ns	101 (25.6%)
Chronic kidney disease	71 (47.3%)	47 (48%)	68 (46.3%)	ns	186 (47.1%)
Cerebrovascular disease	32 (21.9%)	24 (24.2%)	27 (18.4%)	ns	83 (21.2%)

All results presented as n (%) or mean±standard deviation.

Abbreviation: ns, not significant.

**Table 2.** Clinical and Anatomical Characteristics of the Study Group.

	Surgery (n=150)	Hybrid (n=98)	Endovascular (n=147)	p	Total (n=395)
Time since symptom onset					
Less than 6 hours	43 (28.7%)	26 (26.5%)	45 (30.6%)	<0.001	114 (28.9%)
6 to 24 hours	58 (38.7%)	35 (35.7%)	23 (15.6%)		116 (29.4%)
24 to 48 hours	13 (8.7%)	11 (11.2%)	18 (12.2%)		42 (10.6%)
48 hours to 2 weeks	36 (24.0%)	26 (26.5%)	61 (41.5%)		123 (31.1%)
Rutherford ALI classification					
Stadium I	1 (0.7%)	2 (2.0%)	21 (14.3%)	<0.001	24 (6.1%)
Stadium IIA	26 (17.3%)	24 (24.5%)	74 (50.3%)		124 (31.4%)
Stadium IIB	81 (54.0%)	47 (48.0%)	47 (32.0%)		175 (44.3%)
Stadium III	42 (28.0%)	25 (25.5%)	5 (3.4%)		72 (18.2%)
Previous interventions in the index limb	43 (29.1%)	40 (40.4%)	82 (55.8%)	<0.001	165 (41.9%)
Thrombosis in situ (atherosclerotic)	53 (35.6%)	39 (39.4%)	61 (41.5%)	ns	153 (38.7%)
Embolization	86 (57.7%)	51 (51.5%)	44 (29.9%)	<0.001	181 (45.8%)
Bare-metal-stent occlusion	6 (4.0%)	6 (6.1%)	31 (21.1%)	<0.001	43 (10.9%)
Drug-eluting-stent occlusion	1 (0.7%)	0	6 (4.1%)	0.026	7 (1.8%)
Stent-graft occlusion	4 (2.7%)	10 (10.2%)	5 (3.4%)	0.016	19 (4.8%)
Autologous bypass occlusion	2 (1.3%)	2 (2.0%)	4 (2.7%)	ns	8 (2.0%)
Synthetic bypass occlusion	22 (14.7%)	16 (16.3%)	24 (16.3%)	ns	62 (15.7%)

All results presented as n (%).

Abbreviations: ALI, acute limb ischemia; ns, not significant.

Acute limb ischemia was defined as the sudden decrease of blood inflow to the lower extremity lasting for less than 14 days upon presentation. Clinical severity of the condition was categorized according to the Rutherford classification as follows: viable (I), marginally threatened (IIa), immediately threatened (IIb), and irreversible (III).^16^

The primary endpoint of this study was defined as amputation-free survival (AFS), the composite endpoint of either major (above the ankle) amputation or death, whichever occurred first.

Secondary endpoints were defined as overall survival, amputation-free time, defined as time after the index procedure without major amputation, and reintervention-free time during the follow-up. Moreover, perioperative outcomes during the first 30 days after the procedure were also controlled and reported, including access complications (bleeding, wound infection, and pseudoaneurysm), brain injury classified as either ischemic (major stroke or transitory ischemic attack [TIA]) or intracranial bleeding, reintervention of any kind, major amputation, the composite endpoint of MACCE (major adverse cardiac and cerebrovascular event) and acute kidney injury (AKI). Acute kidney injury was classified according to the KDIGO criteria.^17^ A list of all measured 30-day outcomes can be found in Table 3.

**Table 3.** Outcomes in the First 30 Days.

Outcome	Surgery (n=150)	Hybrid (n=98)	Endovascular (n=147)	p	Total (n=266)
Death	25 (16.8%)	14 (14.1%)	5 (3.4%)	<0.001	44 (11.1%)
Amputation	14 (9.4%)	10 (10.1%)	9 (6.1%)	ns	33 (8.4%)
Reintervention	41 (27.5%)	26 (26.3%)	36 (24.5%)	ns	103 (26.1%)
MACCE	16 (10.7%)	14 (14.1%)	8 (5.4%)	ns	38 (9.6%)
Stroke/TIA	5 (3.4%)	1 (1%)	1 (0.7%)	ns	7 (1.8%)
Re-occlusion	28 (18.8%)	14 (14.1%)	12 (8.2%)	0.029	54 (13.7%)
Wound infection	15 (10.1%)	10 (10.1%)	4 (2.7%)	0.025	29 (7.3%)
Distal embolization	2 (1.3%)	4 (4%)	15 (10.2%)	0.003	21 (5.3%)
Acute kidney injury	2 (1.3%)	3 (3%)	2 (1.4%)	ns	7 (1.8%)
Intracranial bleeding	3 (2%)	0	2 (1.4%)	ns	5 (1.3%)

All results presented as n (%).

Abbreviations: MACCE, major adverse cardiac and cerebrovascular events; ns, not significant; TIA, transient ischemic attack.

### Statistical Analysis

Continuous data were reported as the mean±standard deviation or median with the respective value range. Categorical data were expressed as absolute numbers and % prevalence the study cohort. Categorical variables were compared using the χ^2^ test or the Fisher exact test for discrete values. Independent 2-sample *t* tests were used for normally distributed continuous variables and the Wilcoxon rank-sum test was used for non-normally distributed continuous and ordinal variables. Proportional hazards’ modeling was used to assess for independent risk factors for each endpoint while controlling for possible confounders. Survival curves were constructed using the Kaplan-Meier estimator to estimate the AFS time, overall survival time, amputation-free time, and reintervention-free time. All tests were 2-sided. Missing values were ignored when less than 2% of the values of each variable were missing. A p value of <0.05 was considered statistically significant. All statistical analyses were performed using SPSS version 26.0 for Windows (IBM Corp, Armonk, NY).

## Results

During the study period 395 patients (mean age=71.1±13.6 years; 48.9% males) with ALI underwent revascularization and were included in the study. No variables had more than 2% of their values missing, so that, no imputation was needed. Surgery was preferred in 38% of the cases (150/395), HT in 24.8% (98/395), and ET in 37.2% (147/395) of the cases. Patients treated by endovascular means were younger than those treated surgically (mean age, ET: 69±12.5 years vs ST: 73.1±14.8, p=0.035), whereas surgical patients suffered more often from atrial fibrillation (ST: 39.6%; 59/150 vs ET: 21.1%; 31/147, p=0.001) and active malignancy (ST: 29.6%; 39/395 vs ET: 11.6%; 17/147, p=0.004) compared with patients treated with endovascular techniques. An overview of the patient demographics and comorbidities can be found in Table 1.

Significantly, more patients presenting with symptoms lasting less than 24 hours underwent ST or HT (ST: 67.4% vs HT: 62.2% vs ET: 46.2%, p<0.001), as opposed to most patients with a symptom onset of more than 48 hours prior to presentation undergoing primarily endovascular revascularization (ST: 24% vs HT: 26.5% vs ET: 41.5%, p<0.001). Most patients presenting with a Rutherford Stadium IIa ischemia were treated by endovascular means (Rutherford IIa: ST: 17.3% vs HT: 24.5% vs ET: 50.3%, p<0.001), whereas in Rutherford Stadium IIb and III cases, mostly surgical or hybrid revascularization was undertaken (Rutherford IIb and III: ST: 82% vs HT: 73.5% vs ET: 35.4%, p<0.001). Moreover, most of the patients undergoing endovascular repair had a previous vascular intervention in the index limb compared with surgical patients (ST: 29.1% vs ET: 55.8%, p<0.001). A detailed presentation of clinical characteristics can be found in Table 2.

Catheter-directed thrombolysis as a sole procedure was performed in 25.2% (37/147) of the cases in the ET group, CDT in conjunction with endovascular thrombectomy was used in 24.5% (36/147) and endovascular thrombectomy alone in 50.3% of the cases (74/147). The type of endovascular thrombectomy performed was rotational in 30.6% (45/147), pharmacomechanical rheolytic in 25.2% (37/147), continuous aspiration in 17.7% (26/147), and mechanical/manual in 1.4% (2/147) of the cases. In the ST group, 114 patients (76.5%) underwent a transfemoral and 20 patients (13.4%) a transpopliteal thrombectomy. Bypass grafting was performed in 28 cases (18.8%) and a completion angiography in 112 cases (75.2%). In the HT group, 59.2% (58/98) of the procedures were focused on the iliac and common femoral segment.

### Early (30-Day) Outcomes

Mortality in the first 30 days postoperatively was significantly higher in the ST and HT groups compared with ET (ST: 16.8% vs HT: 14.1% vs ET: 3.4%, p<0.001). Moreover, patients treated by ST or HT showed significantly higher re-occlusion (ST: 18.8% vs HT: 14.1% vs ET: 8.2%, p=0.029) and access complications (ST: 10.1% vs HT: 10.1% vs ET: 2.7%, p=0.025) in the early postprocedural period than ET. Patients undergoing ET showed more often distal embolization than in the ST and HT groups (ST: 1.3% vs HT: 4% vs ET: 10.2%, p=0.003). Early perioperative outcomes are demonstrated in Table 3.

### Outcomes During Follow-Up

Median follow-up time was 20 months (range: 0–111 months). Endovascular treatment showed significantly better AFS rates during follow-up compared with ST (ST: 60.7% vs ET: 75.5%; HR: 1.89, 95% CI=1.2–2.9, p<0.001) and HT (HT: 61.2% vs ET: 75.5%; HR: 1.73, 95% CI=1.1–3.1, p<0.001). In the multivariate analysis, ST (HR=1.89, 95% CI=1.2–2.9, p<0.001), HT (HR=1.73, 95% CI=1.1–3.1, p<0.001), active malignancy (HR=2.13, 95% CI=1.5–3.1, p<0.001) and patient’s age above 65 years (65–75 vs <65 years, HR=2.36, 95% CI=1.4–4, p<0.001; >75 vs <65 years, HR=3.63, 95% CI=2.2–5.9, p<0.001) were strongly associated with a lower AFS rate. The variables used in the multivariate analysis can be found in (Supplemental Table 1).

In the univariate analysis, mortality during follow-up was significantly lower after ET compared with ST (HR=2.21, 95% CI=1.31–3.74, p=0.003) and HT (HR=2.04, 95% CI=1.17–3.56, p=0.012). In the multivariate analysis, ST and HT were both independently associated with higher mortality rates compared with ET. Coronary heart disease, age above 65 years, chronic kidney disease and malignancy were also delineated as independent risk factors for higher mortality in the multivariate analysis (Supplemental Table 2).

Amputation rates during follow-up were lower after ET compared with ST in the univariate analysis (HR=2.27, 95% CI=1.19–4.35, p=0.013) and comparable with HT (HR=2.00, 95% CI=0.98–4.06, p=0.055). In the multivariate analysis, however, there was no statistically significant association between the type of repair and amputation rates, Rutherford III ischemia was the only risk factor independently associated with higher amputation rates (Supplemental Table 3). Reintervention rates did not differ between the groups in the univariate analysis (ET vs ST: HR=1.52, 95% CI=0.99–2.31, p=0.053; ET vs HT: HR=1.3, 95% CI=0.81–2.07, p=0.27). Rutherford III ischemia was delineated as an independent risk factor associated with increased reintervention rates, whereas symptom duration of less than 6 hours before the index treatment showed a protective effect against reintervention in the multivariate analysis (Supplemental Table 4).

**Figure 1.**
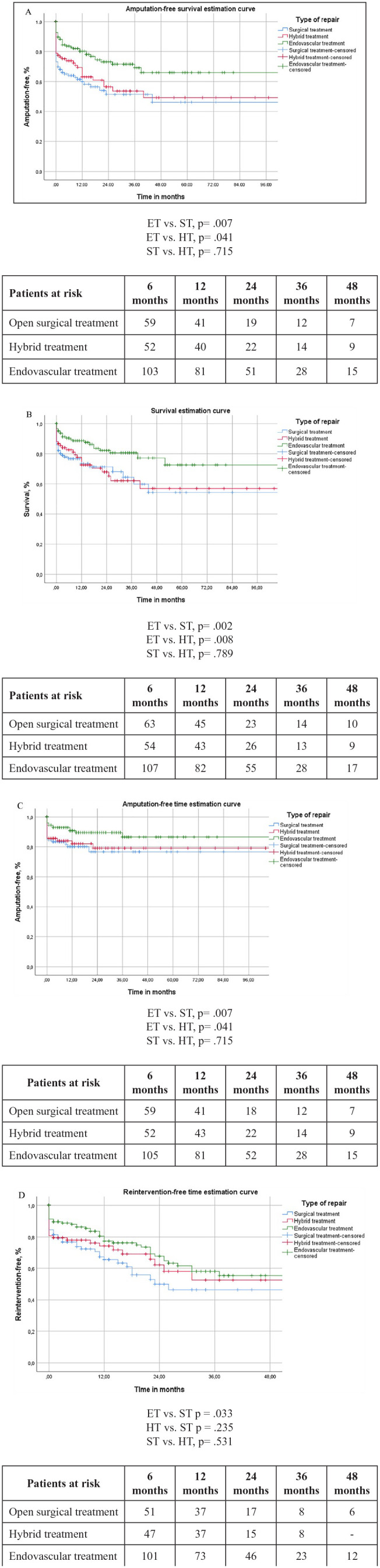
(A) Kaplan-Meier amputation-free survival estimation curve. All results represented are within a standard error of 10%. (B) Kaplan-Meier survival estimation curve. All results represented are within a standard error of 10%. (C) Kaplan-Meier amputation-free time estimation curve. All results represented are within a standard error of 10%. (D) Kaplan-Meier reintervention-free time estimation curve. All results represented are within a standard error of 10%.

The respective Kaplan-Meier estimation curves are demonstrated in Figures 1A–D.

## Discussion

Acute limb ischemia remains a potentially debilitating disease with increased mortality and major amputations rates.^18^ Despite the technical advancements in this field, there is still a relevant lack of data on contemporary devices, medications, and techniques. Most reports in the last years evaluated either a small cohort,^9^ a single treatment option without control group,^18^ or reported only on the peri-interventional outcomes.^13^ The current analysis, which assessed all currently available treatment options, showed a significantly lower mortality and morbidity rates at 30 days following endovascular therapy, although the early amputation rates did not differ significantly between the 3 groups. Moreover, ET was associated with improved AFS and mortality during follow-up, although the reintervention and major amputation rates were comparable with hybrid and surgical therapy.

Endovascular therapy is the first-line treatment option for most patients with chronic ischemia of the lower extremities.^19,20^ The minimally invasive nature of percutaneous procedures offers a benefit compared with open repair especially in patients with increased comorbidity. Despite the promising results of the initial randomized trials, the increased risk for bleeding, the need for ICU stay and the delayed reperfusion of the affected limb did not allow a similar adoption of an endovascular first approach for most patients with ALI.^11^ However, the development of endovascular thrombectomy techniques enabled the rapid clot removal and reperfusion of the lower extremities, while reducing the need for CDT and accordingly of ICU stay. In this cohort, more than 50% of patients treated by endovascular thrombectomy devices did not require a CDT. Similarly, in the prospective PEARL registry (PEripheral Use of Angiojet Rheolytic Thrombectomy), the use of PMT reduced the need for CDT in half of the procedures.^21^ This might be crucial in patients with increased risk for bleeding complications as well as in cases of severe ischemia of the limb. Of note, although current recommendations suggest a rapid restoration of the blood flow with thrombus extraction and thrombo-aspiration, especially in patients with neurologic deficit, recent studies showed that the primary mode of endovascular therapy remains CDT.^22,23^ The updated European guidelines for the treatment of ALI among patients with COVID-19 infection also highlighted the importance of endovascular thrombectomy, given the limited resources in ICU and high dependency units during the pandemic.^22^

The selection of the used thrombectomy devices should be based on the nature (embolic vs thrombotic) and the location (tibial, femoropopliteal, iliac) of the disease as not all modalities show the same efficacy in the treatment of different pathologies. Although the literature is not conclusive, 2 recent observational studies reported a benefit from ET in patients with ALI. Argyriou et al^24^ reported a higher AFS after endovascular revascularization for patients with ALI and active neoplastic disease, while the ENSUPRO (ENdovascular versus SUrgical Treatment of All-comer Patients With PROsthetic Bypass Graft Occlusion) registry reported the same finding for patients with prosthetic bypass occlusion.^25^ Both of these reports, like our analysis, included patients treated by endovascular thrombectomy devices and not only CDT.^24,25^

For many decades, surgical embolectomy was the main revascularization strategy for ALI, while bypass grafting was indicated in cases of unsuccessful restoration of the limb perfusion. Although primary conventional thrombectomy might be particularly helpful for embolic occlusion of the groin vessels or the popliteal artery, this can be challenging in cases of preexisting peripheral atherosclerosis.^26^ In these lesions, ALI is usually the result of a plaque rupture and thrombus propagation, which leads to an acute vessel occlusion. A Fogarty-based embolectomy might then lead to long, flow-limiting dissections, which will require adjunctive procedures. In this context, current recommendations suggest the use of guidewire-based thrombectomy as well as a final angiographic imaging.^12^ Notably, in our analysis less than half of the acute arterial occlusions were the result of embolization and almost 55% of patients had atherosclerotic disease with an acute-on-chronic occlusion. A retrospective analysis of patients treated within the Veterans Affairs Healthcare System, observed also a very high prevalence (75%) of co-existing peripheral arterial disease in patients with ALI.^27^ However, other observational studies found a higher prevalence of arterial thromboembolism, whereas native vessel thrombosis, graft occlusion, and popliteal artery aneurysm were less frequently the cause of ALI.^22^

Furthermore, given the inhomogeneous nature of HT, the evaluation of this treatment group remains a challenge. These patients either present with multi-level disease and endovascular techniques are used to improve the inflow or outflow of a surgical reconstruction or they are used as bail-out procedures (eg, dissection after embolectomy, aspiration for embolization, etc). Accordingly, these patients quite often represent a very high-risk subgroup. Nonetheless, in this study the outcomes of HT did not differ significantly from the performance of open repair.

Finally, increased patient age as well as the presence of severe coronary disease, chronic kidney disease, and active malignancy were identified as independent predictors of lower AFS rates. Of note, active neoplastic disease was previously considered a contraindication for CDT. However, current European guidelines suggest the revascularization of selected patients with malignancy, while CDT can also be applied in these patients with acceptable bleeding risk profile.^12^ Again, the use of thrombectomy devices might be beneficial in these high-risk patients and reduce the observed morbidity and mortality.^24^ A recent epidemiological study from Sweden also found a significantly lower AFS among older patients, while all patients living in a nursing home on admission either died or underwent a major amputation 12 months after the initial admission.^22^

### Limitations

This study has several limitations, primarily due to its retrospective nature. As in every retrospective cohort, an inherent risk of bias is present, which we tried to mitigate by including all consecutive patients treated. This leads to heterogeneity in the patient populations limiting the generalizability of the results, although it could be argued that it also reflects real-world scenarios. Nonetheless, only prospective or, ideally, blinded randomized trials could provide a definitive solution to this problem. Moreover, the small number of sole CDT procedures in this cohort did not allow for an adequately powered subgroup analysis against other treatment methods.

## Conclusions

Endovascular treatment of patients with ALI was an independent predictor of higher AFS rates compared with ST and HT. Endovascular therapy was also associated with lower early mortality rates, increased overall survival, and increased amputation-free time during follow-up. A large prospective or randomized trial using contemporary techniques and medications could potentially validate these results.

## Supplemental Material

sj-docx-1-jet-10.1177_15266028231210232 – Supplemental material for Outcomes After Open Surgical, Hybrid, and Endovascular Revascularization for Acute Limb Ischemia Supplemental material, sj-docx-1-jet-10.1177_15266028231210232 for Outcomes After Open Surgical, Hybrid, and Endovascular Revascularization for Acute Limb Ischemia by Nikolaos Konstantinou, Angeliki Argyriou, Felicitas Dammer, Theodosios Bisdas, Gregory Chlouverakis, Giovanni Torsello, Nikolaos Tsilimparis and Konstantinos Stavroulakis in Journal of Endovascular Therapy

sj-docx-2-jet-10.1177_15266028231210232 – Supplemental material for Outcomes After Open Surgical, Hybrid, and Endovascular Revascularization for Acute Limb Ischemia Supplemental material, sj-docx-2-jet-10.1177_15266028231210232 for Outcomes After Open Surgical, Hybrid, and Endovascular Revascularization for Acute Limb Ischemia by Nikolaos Konstantinou, Angeliki Argyriou, Felicitas Dammer, Theodosios Bisdas, Gregory Chlouverakis, Giovanni Torsello, Nikolaos Tsilimparis and Konstantinos Stavroulakis in Journal of Endovascular Therapy

sj-docx-3-jet-10.1177_15266028231210232 – Supplemental material for Outcomes After Open Surgical, Hybrid, and Endovascular Revascularization for Acute Limb Ischemia Supplemental material, sj-docx-3-jet-10.1177_15266028231210232 for Outcomes After Open Surgical, Hybrid, and Endovascular Revascularization for Acute Limb Ischemia by Nikolaos Konstantinou, Angeliki Argyriou, Felicitas Dammer, Theodosios Bisdas, Gregory Chlouverakis, Giovanni Torsello, Nikolaos Tsilimparis and Konstantinos Stavroulakis in Journal of Endovascular Therapy

sj-docx-4-jet-10.1177_15266028231210232 – Supplemental material for Outcomes After Open Surgical, Hybrid, and Endovascular Revascularization for Acute Limb Ischemia Supplemental material, sj-docx-4-jet-10.1177_15266028231210232 for Outcomes After Open Surgical, Hybrid, and Endovascular Revascularization for Acute Limb Ischemia by Nikolaos Konstantinou, Angeliki Argyriou, Felicitas Dammer, Theodosios Bisdas, Gregory Chlouverakis, Giovanni Torsello, Nikolaos Tsilimparis and Konstantinos Stavroulakis in Journal of Endovascular Therapy
