# Supplementary material for: Outcomes After Open Surgical, Hybrid, and Endovascular Revascularization for Acute Limb Ischemia
Source: J Endovasc Ther. 2023 Nov 27;32(5):1499–507. doi: 10.1177/15266028231210232 (PMC12433533; doi:10.1177/15266028231210232)
Supplement: sj-docx-1-jet-10.1177_15266028231210232 – Supplemental material for Outcomes After Open Surgical, Hybrid, and Endovascular Revascularization for Acute Limb Ischemia [file sj-docx-1-jet-10.1177_15266028231210232.docx]

| Covariate | B | SE | Wald | Sig. | HR | 95% CI |
| --- | --- | --- | --- | --- | --- | --- |
| Age <65 years (reference) |  |  | 26.94 | < 0.001 |  |  |
| Age 65-75 years | 0.857 | 0.269 | 10.13 | 0.001 | 2.36 | 1.39 to 4.00 |
| Age >75 years | 1.29 | 0.25 | 26.45 | < 0.001 | 3.63 | 2.22 to 5.94 |
| Malignancy | 0.76 | 0.19 | 16.29 | < 0.001 | 2.14 | 1.48 to 3.09 |
| Endovascular treatment (reference) |  |  | 9.44 | 0.009 |  |  |
| Surgical treatment | 0.64 | 0.22 | 8.77 | 0.003 | 1.89 | 1.24 to 2.89 |
| Hybrid treatment | 0.55 | 0.23 | 5.56 | 0.018 | 1.74 | 1.10 to 2.75 |

**Table 4: Multivariate analysis of protective or risk increasing factors for major amputation and/or death**
