# Supplementary material for: Outcomes After Open Surgical, Hybrid, and Endovascular Revascularization for Acute Limb Ischemia
Source: J Endovasc Ther. 2023 Nov 27;32(5):1499–507. doi: 10.1177/15266028231210232 (PMC12433533; doi:10.1177/15266028231210232)
Supplement: sj-docx-2-jet-10.1177_15266028231210232 – Supplemental material for Outcomes After Open Surgical, Hybrid, and Endovascular Revascularization for Acute Limb Ischemia [file sj-docx-2-jet-10.1177_15266028231210232.docx]

| Covariate | B | SE | Wald | Sig. | HR | 95% CI |
| --- | --- | --- | --- | --- | --- | --- |
| Age <65 years (reference) |  |  | 26.94 | 0.001 |  |  |
| Age 65-75 years | 1.12 | 0.41 | 7.47 | 0.006 | 3.06 | 1.37 to 6.82 |
| Age >75 years | 1.45 | 0.39 | 13.66 | < 0.001 | 4.26 | 1.98 to 9.19 |
| Coronary heart disease | 0.58 | 0.22 | 6.74 | 0.009 | 1.79 | 1.15 to 2.77 |
| Chronic kidney disease | 0.73 | 0.24 | 9.25 | 0.002 | 2.07 | 1.30 to 3.32 |
| Malignancy | 0.93 | 0.23 | 16.53 | < 0.001 | 2.52 | 1.62 to 3.94 |
| Endovascular treatment (reference) |  |  | 6.02 | 0.049 |  |  |
| Surgical treatment | 0.59 | 0.27 | 4.68 | 0.031 | 1.80 | 1.06 to 3.06 |
| Hybrid treatment | 0.63 | 0.29 | 4.84 | 0.028 | 1.88 | 1.07 to 3.29 |

**Multivariate analysis of protective or risk increasing factors for death**
