# Supplementary material for: Outcomes After Open Surgical, Hybrid, and Endovascular Revascularization for Acute Limb Ischemia
Source: J Endovasc Ther. 2023 Nov 27;32(5):1499–507. doi: 10.1177/15266028231210232 (PMC12433533; doi:10.1177/15266028231210232)
Supplement: sj-docx-3-jet-10.1177_15266028231210232 – Supplemental material for Outcomes After Open Surgical, Hybrid, and Endovascular Revascularization for Acute Limb Ischemia [file sj-docx-3-jet-10.1177_15266028231210232.docx]

| Covariate | B | SE | Wald | Sig. | HR | 95% CI |
| --- | --- | --- | --- | --- | --- | --- |
| Symptom duration between 6 and 24 hours | 0.41 | 0.28 | 2.23 | 0.135 | 1.51 | 0.88 to 2.58 |
| Rutherford Stadium III | 1.23 | 0.29 | 17.54 | < .001 | 3.41 | 1.92 to 6.05 |
| Endovascular treatment (reference) |  |  | 0.45 | 0.80 |  |  |
| Surgical treatment | 0.25 | 0.37 | 0.44 | 0.51 | 1.28 | 0.62 to 2.64 |
| Hybrid treatment | 0.20 | 0.39 | 0.25 | 0.61 | 1.22 | 0.57 to 2.61 |

**Multivariate analysis of protective or risk increasing factors for major amputation**
