# Supplementary material for: Outcomes After Open Surgical, Hybrid, and Endovascular Revascularization for Acute Limb Ischemia
Source: J Endovasc Ther. 2023 Nov 27;32(5):1499–507. doi: 10.1177/15266028231210232 (PMC12433533; doi:10.1177/15266028231210232)
Supplement: sj-docx-4-jet-10.1177_15266028231210232 – Supplemental material for Outcomes After Open Surgical, Hybrid, and Endovascular Revascularization for Acute Limb Ischemia [file sj-docx-4-jet-10.1177_15266028231210232.docx]

| Covariate | B | SE | Wald | Sig. | HR | 95% CI |
| --- | --- | --- | --- | --- | --- | --- |
| Symptom duration less than 6 hours | -0.50 | 0.24 | 4.5 | 0.034 | 0.61 | 0.38 to 0.96 |
| Rutherford Stadium III | 0.67 | 0.23 | 8.54 | 0.003 | 1.96 | 1.25 to 3.08 |
| Endovascular treatment (reference) |  |  | 0.41 | 0.816 |  |  |
| Surgical treatment | 0.15 | 0.24 | 0.40 | 0.529 | 1.16 | 0.73 to 1.85 |
| Hybrid treatment | 0.06 | 0.25 | 0.06 | 0.804 | 1.07 | 0.65 to 1.75 |

**Multivariate analysis of protective or risk increasing factors for reintervention**
